# Draft Genome Sequence of Bradyrhizobium elkanii Strain SEMIA 938, Used in Commercial Inoculants for *Lupinus* spp. in Brazil

**DOI:** 10.1128/MRA.00546-19

**Published:** 2019-07-11

**Authors:** Mariangela Hungria, Jakeline Renata Marçon Delamuta, Renan Augusto Ribeiro, Marco Antonio Nogueira

**Affiliations:** a Embrapa Soja, Soil Biotechnology Laboratory, Londrina, Paraná, Brazil; b CNPq, SHIS QI 1 Conjunto B, Brasília, Federal District, Brazil; Georgia Institute of Technology

## Abstract

Due to its high capacity for nitrogen fixation, strain SEMIA 938 is used in commercial inoculants for lupins in Brazil. Its genome was estimated at 8,780,064 bp and indicates that it belongs to the Bradyrhizobium elkanii species, while the analysis of nodulation genes classifies the strain in the symbiovar sojae.

## ANNOUNCEMENT

Biological nitrogen fixation (BNF) is a key process for global N balance and N input in agriculture (1). Outstanding rates of BNF have been cited for grain crops such as soybean (Glycine max) (2); however, contributions of forage legumes and green manures also impact agriculture sustainability and mitigation of greenhouse gases (1, 3). The genus *Lupinus* comprises more than 200 species of subtropical and temperate annual herb legumes used for grain consumption, green manure, and pastures; the majority of the species are indigenous to the Americas. In Brazil, the area with lupins is still modest, cropped mainly with European species in the southern states. For the past 3 decades, strain SEMIA 938, originally isolated from a nodule of Lupinus albus inoculated with a commercial inoculant for *Lupinus* from the United States (Hansen), has been officially authorized by Brazilian legislation for use in commercial inoculants for *L. albus* and Lupinus angustifolius (4). However, few studies have been performed with strain SEMIA 938, and now we describe its sequenced genome.

Strain CNPSo 938 (= CNPSo 1004) was grown in modified yeast mannitol (YM) medium at 28°C for 5 days (5), and then total DNA was extracted using the DNeasy blood and tissue kit (Qiagen) and processed on the MiSeq platform (Illumina) at Embrapa Soja. Genome sequencing resulted in 871,756,030 bp with assemblage and quality control performed with the A5-MiSeq pipeline (*de novo*) v.20140604. Sequencing allowed 99.29-fold genome coverage in 80 contigs with an *N*_50_ value of 279,572 bp. Using QUAST v.2.0 (6) with default parameters, the genome was estimated at 8,780,064 bp with a G+C content of 63.87%. A total of 8,821 coding DNA sequences (CDSs) were identified using Rapid Annotations using Subsystems Technology (RAST) (7), with 49% classified in 381 subsystems, and the annotation is available in the GenBank database.

Average nucleotide identity (ANI) was successfully used for taxonomic purposes, with cutoff values of 95 to 96% corresponding to 70% DNA-DNA hybridization (DDH), the threshold for species delineation (8). Using the ANI calculator (9) for comparison with type strains of all *Bradyrhizobium* species, the highest identity (95.94%) of SEMIA 938 was with Bradyrhizobium elkanii USDA 76^T^, while digital DDH (dDDH), evaluated with the Genome-to-Genome Distance Calculator (GGDC) v.2.1 using the recommended “formula 2” (10), resulted in a value of 65.50%. Therefore, although SEMIA 938 shows the highest relatedness with *B. elkanii*, it might represent a new species.

Nodulation (*nod*) genes of *Bradyrhizobium* species are key for the BNF process with legumes (11, 12). In SEMIA 938, *nod* genes are organized as in Bradyrhizobium diazoefficiens strain USDA 110^T^, with the regulatory *nodD1* and *nodD2* genes followed by the operon *nodABC*. The genome also carries *nodZ*, an unusual *nod* gene related to host specificity with constitutive expression independent of *nodD* (11, 13). To determine the symbiovar, or biological variant of symbiotic genes (14), of SEMIA 938, *nodC*, *nodY/K*, and *nifH* were analyzed, confirming that the strain belongs to the symbiovar sojae, recently described by our research group (12). Information available from this genome helps to elucidate the phylogenetics of conserved and nitrogen-fixation genes of the *Bradyrhizobium* genus.

### Data availability.

This whole-genome shotgun project has been deposited at DDBJ/EMBL/GenBank under the GenBank accession number SZZP00000000, BioProject number PRJNA541436, and BioSample number SAMN11585296. The version described in this paper is SZZP01000000.
